# Biogenesis aberration: One of the mechanisms of thrombocytopenia in COVID-19

**DOI:** 10.3389/fphys.2023.1100997

**Published:** 2023-03-20

**Authors:** Cuiting Shan, Feng Yu, Xuemei Deng, Li Ni, Xuming Luo, Jialin Li, Si Cai, Mian Huang, Xiongbiao Wang

**Affiliations:** ^1^ Department of Respiratory Medicine, Putuo Hospital, Shanghai University of Traditional Chinese Medicine, Shanghai, China; ^2^ Shanghai Putuo District People’s Hospital, Shanghai, China; ^3^ Department of Neurology, Wuhan Third Hospital, China and Tongren Hospital of Wuhan University, Wuhan, Hubei, China

**Keywords:** COVID-19, platelet, ARDS, platelet production, IPF

## Abstract

**Background:** The pathogenesis of COVID-19, including thrombocytopenia, has not been fully clarified. The lungs are a major organ of platelet production and thrombocytopenia induced by severe COVID-19 was proposed.

**Methods:** the change of platelet level was analysed with clinical parameters in 95 hospitalized COVID-19 patients in Wuhan Third Hospital. The production of platelets in the lungs was explored in an ARDS rat model.

**Results:** The level of platelets was negatively correlated with disease severity and was recovered with disease improvement. The non-survivors were accompanied by lower levels of platelet. The odds ratio (OR) of the valley level of the platelet count (PLTlow) was greater than 1, suggesting that PLTlow could be a death exposure factor. The platelet/lymphocyte ratio (PLR) was positively associated with severity of COVID-19, and the platelet/lymphocyte ratio threshold of 248.5 was best correlated with death risk (sensitivity 0.641 and specificity 0.815). To demonstrate the possible biogenesis aberration of platelet in lungs, an LPS-induced ARDS rat model was applied. Lower level of platelet in peripheral and less production of platelet from lungs in ARDS were demonstrated. Though megakaryocyte (MK) number in ARDS lungs is higher than controls, the immature platelet fraction (IPF) in postpulmonary blood is still at the same level as prepulmonary in ARDS rat, indicating that ARDS rats generated fewer platelets in lungs.

**Conclusion:** Our data suggested that COVID-19-induced severe lung inflammation may impair platelet production in the lung. Thrombocytopenia may be mainly caused by platelet consumption for multiorgan thrombosis; however, biogenesis aberration of platelet in the lung induced by diffuse interstitial pulmonary damage cannot be ruled out.

## Introduction

A new viral pneumonia exploded in early 2020, and the pathogen SARS-COV-2 was quickly identified. COVID-19 was designated by the World Health Organization at the same time. The characteristics of the virus and clinical features of the disease are being revealed more clearly. SARS-COV-2 is an RNA virus and can propagate by droplets. The most common symptoms are fever, fatigue, cough, and dyspnoea. Laboratory abnormalities included normal or decreased white blood cell count, decreased lymphocyte count, elevated C-reactive protein (CRP), increased liver enzymes, lactate dehydrogenase (LDH), creatine kinase, and myoglobin in some patients. In severe cases, CRP, erythrocyte sedimentation rate, LDH, D-dimer, creatinine, cardiac troponin I, alanine aminotransferase, aspartate aminotransferase, and prothrombin time were elevated more significantly.

As platelet plays multiple functions in haemostasis, thrombosis, inflammation, and fibrosis, it has received attention since COVID-19 early prevalence. The features of platelets are affected by SARS-COV-2, and thus, platelets play roles in the pathogenesis of COVID-19. Thrombocytopenia has been reported as a predictor for disease severity or a worse prognosis (Lippi et al., 2020) (Yang X. et al., 2020) (Mo et al., 2021), although several studies have not found a correlation between platelet levels and disease severity or mortality (Fan et al., 2020) (Terpos et al., 2020). Beside of the lower number, there were unique features of platelets in COVID-19 patients, including larger, more granular and a higher mean platelet volume (MPV) (Taha et al., 2021) (Zhang et al., 2020). The function of platelets from COVID-19 patients was abnormal, with hyperresponsiveness to agonist stimulation, and interacted more with leukocytes at baseline, indicating crosstalk between platelets and leukocytes during SARS-CoV-2 infection (Zhang et al., 2020) (Manne et al., 2020). Therefore, platelet alterations are involved in COVID-19 in number, morphology, and function or “platelet heterogeneity” (van der Meijden and Heemskerk, 2019).

Recent studies on the pathogenesis of platelets in COVID-19 mainly include the following: 1. As platelets express ACE2 and TMPRSS2, platelets can be activated by SARS-CoV-2 directly through the S protein (Zhang et al., 2020). However, there are contrary data that platelets do not express ACE2, and SARS-COC-2 virus could not be detected within platelets from COVID-19 patients (Manne et al., 2020). 2. SARS-CoV-2 may affect the integrity of the vascular endothelium by binding with ACE2 and promote platelet activation and thrombosis by binding with platelets. Varga et al. also reported inflammatory reactions of the endothelium in various organs (lung, heart, small intestine) (Varga et al., 2020); however, the finding was not reproducible in another histologically examined cases (Edler et al., 2020). 3. A recent study showed that COVID-19 significantly altered platelet gene expression, triggering robust platelet hyperreactivity (Manne et al., 2020). 4. Mitochondrial dysfunction leads to a reduced ability of platelets to become procoagulant (Denorme et al., 2020). All these studies indicated platelet plays a very important role in COVID-19.

As the variance reports of platelet count and its pathogenesis in COVID-19 has not been fully clarified, we retrospectively analysed the data from WuHan Third Hospital, a designated temporary COVID-19 hospital. As the lung is a major organ for platelet production (Lefrancais and Looney, 2019), the effect of COVID-19-induced diffuse alveolar damage (DAD) on platelet biogenesis is concerned. An ARDS model was applied to confirm that DAD reduced platelet biogenesis in the lungs.

## Results

### Demographic characteristics

General patient information is present in Tables 1, 2. Of the 95 patients with COVID-19, 49 were male. The age difference among the four groups of disease severity was statistically significant (*p* < 0.05), and the age of critical patients were greater than those with mild and common patients (Table 1). Fourteen patients (14.7%) died, including eight males (57.1%). The age difference between the survived and non-survived patients was also statistically significant (*p* < 0.05). The average age of non-survived patients was 69.93 years old, which was significantly higher than that of the non-survived patients (57.79 years old) (Table 2). There was no difference in gender composition among the groups.

**TABLE 1:** Demographic characteristics and laboratory parameters of patients of different severities.

	Mild	Common	Severe	Critical	*p*-Value
	(*n* = 24)	(*n* = 25)	(*n* = 25)	(*n* = 21)	
Age, years	48.25 ± 18.917*	57.40 ± 16.578^#^	64.56 ± 13.932	65.33 ± 12.619*^,#^	0.001
Sex (men)	11	11	13	14	0.422
PLT0,×10^9/L	231.06 ± 62.450	226.20 ± 75.831	198.96 ± 85.687	213.76 ± 115.815	0.251
LC0,×10^9/L	1.7900 ± .67180*^,#^	1.2632 ± .49900^※^	0.8988 ± 0.56016^#^	0.6767 ± 0.34103*^※^	0.000
PLR0	147.63 ± 87.682*	197.72 ± 85.817^#^	302.04 ± 250.687	421.19 ± 432.822*^,#^	0.000
PLTlow,×10^9/L	219.94 ± 62.804*	197.68 ± 68.089	171.24 ± 63.101	155.00 ± 72.532*	0.007
PLRhigh	153.83 ± 96.047*	220.92 ± 92.177^#^	368.64 ± 522.912	546.95 ± 418.976*^,#^	0.000
PLRlow	150.21 ± 90.158*	175.12 ± 128.442^#^	221.64 ± 150.929	288.33 ± 173.604*^,#^	0.007
Ca^2+^0,mmol/L	2.2654 ± 0.13536*^,#^	2.1317 ± 0.10905^※^	2.0617 ± 0.10573^#※^	1.9910 ± 0.19857*^※^	0.000
Ca^2+^low,mmol/L	2.2675 ± 0.11368*^,#^	2.1548 ± 0.08382^※^	2.0861 ± 0.11317^#^	1.9885 ± 0.19214*^,※^	0.000
ΔCa^2+^,mmol/L	−0.0021 ± 0.06072	−0.0195 ± 0.11504	−0.0243 ± 0.12116	0.0025 ± 0.10083	0.724
Ca^2+^lowest, mmol/L	2.2475 ± 0.12878*^,#^	2.0824 ± 0.10059^※^	1.9850 ± 0.10074^#^	1.9239 ± 0.19239*^,※^	0.000
dCa^2+^,mmol/L	0.0179 ± 0.04520*	0.0516 ± 0.07192	0.0721 ± 0.09160*	0.0583 ± 0.07763	0.029
D-dimer, mg/L	1.79 (1.08)*^,#,※^	0.94 (1.15)*	0.86 (0.49)^#^	0.68 (0.55)^※^	0.000
CRP, mg/L	1.11 (6.98)*^,#^	7.73 (31.44)^※▲^	59.75 (129.75)*^※^	66.57 (127.85)^#▲^	0.000

Abbreviations: PLT0, LC0, PLR0, Ca^2+^0, D-dimer and CRP are platelets, lymphocytes, platelet-lymphocyte ratio, calcium ion, D-dimer and C-reactive protein concentration at admission, respectively. PLTlow is the lowest platelet value during hospitalization, respectively. PLRlow is the PLR corresponding to the lowest platelet value during hospitalization. Ca^2+^low is the calcium ion concentration corresponding to the lowest platelet value. ΔCa^2+^ is the difference between the calcium ion concentration on admission and the calcium corresponding to the lowest platelet value. Ca^2+^ lowest is the lowest calcium ion concentration during hospitalization. dCa^2+^ is the difference between the calcium ion concentration at admission and the lowest calcium ion concentration during hospitalization. A *p*-value of <0.05 was considered significant. *p < 0.05, ^#^ *p* < 0.05, ※*p* < 0.05, ^▲^ *p* < 0.05.

**TABLE 2:** Demographic characteristics and laboratory parameters of dead and non-dead patients.

	Dead	Survivor	*p*-Value
	*n* = 14 (14.7%)	*n* = 81 (85.3%)	
Age, years	69.93 ± 12.175	56.79 ± 17.003	0.007
Sex (men)	8 (57.1%)	41 (50.6%)	0.652
PLT0,×10^9/L	215.00 ± 129.204	217.94 ± 76.670	0.326
LC0,×10^9/L	.6064 ± .29749	1.2683 ± .67292	0
PLR0	487.00 ± 516.418	223.01 ± 169.530	0.003
PLTlow,×10^9/L	150.79 ± 76.736	193.15 ± 67.211	0.036
PLRlow	349.43 ± 205.121	181.32 ± 116.997	0

Abbreviations: PLT0, LC0 and PLR0 are platelets, lymphocytes and platelet-lymphocyte ratio at admission, respectively. PLTlow is the lowest platelet value during hospitalization. PLRlow is the PLR corresponding to the lowest platelet value during hospitalization.

### The level of platelets was associated with disease severity

The relationship of platelet levels among the four groups of patients with mild, common, severe, and critical was analysed (Table 1). We analysed the platelet count at admission, but the differences were not statistically significant (*p* > 0.05). The lowest level during hospitalization among the four groups was significantly different, which indicated that alterations in platelet number were more significant during the peak of disease.

### Correlation analysis between platelets and outcome

The chi square test was used to analyse the correlation between low platelet peak values and patient mortality. According to the characteristics of the data, we defined 150×10^9^/L as the limit of thrombocytopenia in this study. The results showed that X^2^ = 9.185, OR = 6.776, 95% CI 2.007-22.885, and the results were statistically significant (*p* < 0.05) (Table 3). To identify the factors that may affect COVID-19 outcome, we conducted the logistic regression analysis. We analysed PLTlow after the adjustment of age and gender. The result showed that PLTlow was an independent factor for COVID-19 patients (Supplementary Table S1).

**TABLE 3:** The analysis of platelet and death association.

	X^2^	*p*	OR	95%CI
PLTLow	9.185	0.002	6.776	2.007–22.885

Abbreviations: PLTlow is the lowest platelet value during hospitalization.

### Patient platelet/lymphocyte ratio (PLR)

**FIGURE 1:**
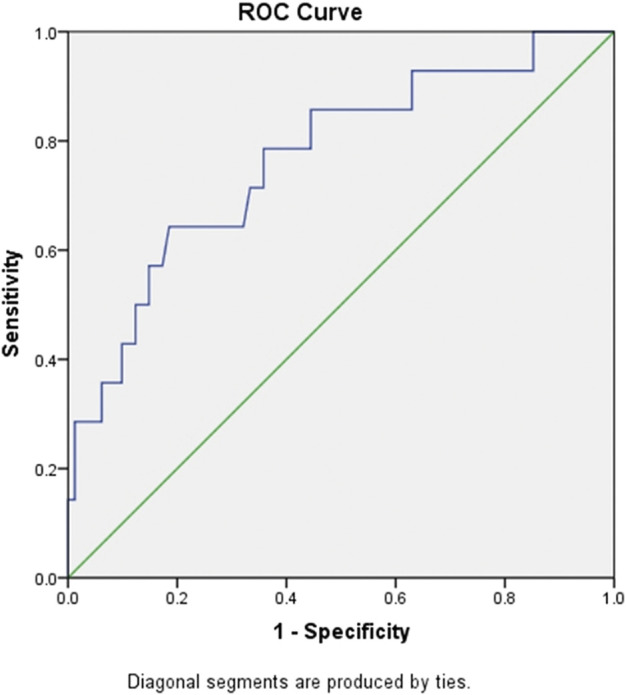
ROC curve of PLR for predicting mortality of in-hospital patients with COVID-19. Abbreviations: ROC, receiver operating characteristic; PLR, platelet/lymphocyte ratio.

PLR is commonly used as an inflammatory index. Here, we also calculated the ratio in the patients. The PLR in the critical patients (421.19) was significantly higher than that in the mild (147.63) and common patients (197.72) at admission. The PLR was significantly different among groups when the platelet count was at the lowest level during hospitalization. PLR was also a sensitive marker for survival. The ROC curve of PLR is shown in Figure 1. The area under the curve (AUC) of PLR was 0.768 (*p* < 0.05), the optimal threshold was 248.5, and the highest sensitivity and specificity were 0.643 and 0.815, respectively, which is consistent with a previous report (Wang C. et al., 2020). For further confirmation, we also analysed PLTlow after the adjustment of age and gender by logistic regression analysis. The result showed that PLRlow was an independent factor for COVID-19 patients (Supplementary Table S1).

### LPS-induced ARDS in rats was confirmed by CT and PaO_2_/FiO_2_ analyses

**FIGURE 2:**
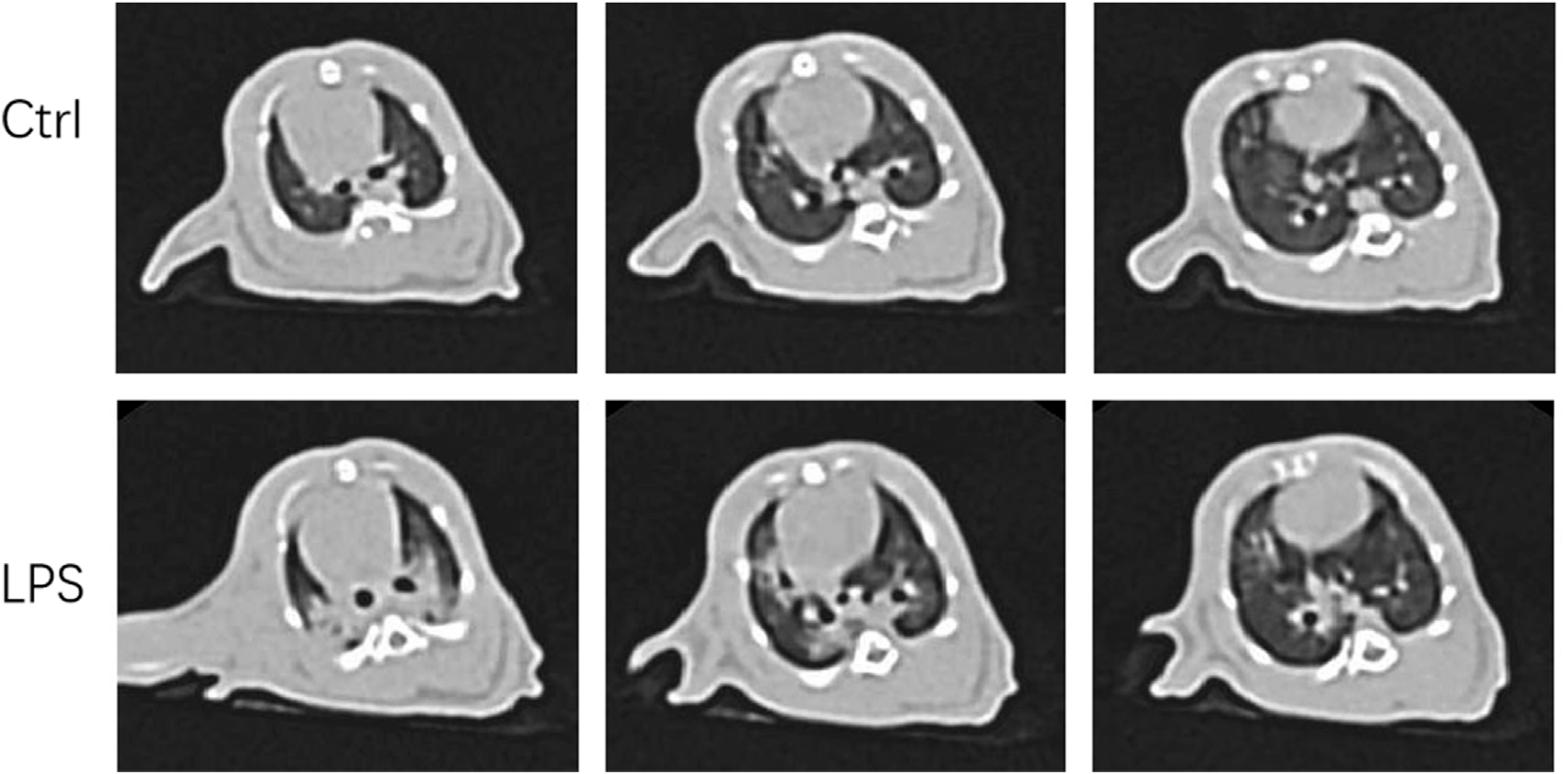
Computer tomography images of LPS-induced ARDS rats and controls. Chest computed tomography was performed in rats induced by LPS or saline (control). Images demonstrate one representative animal per group and were viewed at the same window width (2000 HU) and window level (−500 HU).

We were eager to know if lung injury by COVID-19 affects platelet biogenesis in the lung. Many reports have confirmed that clinical symptoms, chest imaging, and autopsy in COVID-19 are consistent with the pathological process of ARDS (Ma et al., 2020) (Gibson et al., 2020). The ARDS model induced by LPS was used as a substitute for viral challenge because of the biosafety limit. To confirm that the model was successfully established, lung CT was applied. After injection of LPS (20 μg/g body weight) after 12 h, the rats underwent CT scan every 2 hours. At 16 h, lung images of the LPS-induced ARDS group presented blurred and messy bilateral lung markings, multiple consolidation, and patchy shadows with blurry margins (Figure 2), which indicated establishment of the ARDS model. At 24 h, the PaO_2_/FiO_2_ was significantly decreased in LPS-induced ARDS group (Supplementary Figure S1).

### Lung histology of LPS-induced ARDS rat lungs

**FIGURE 3:**
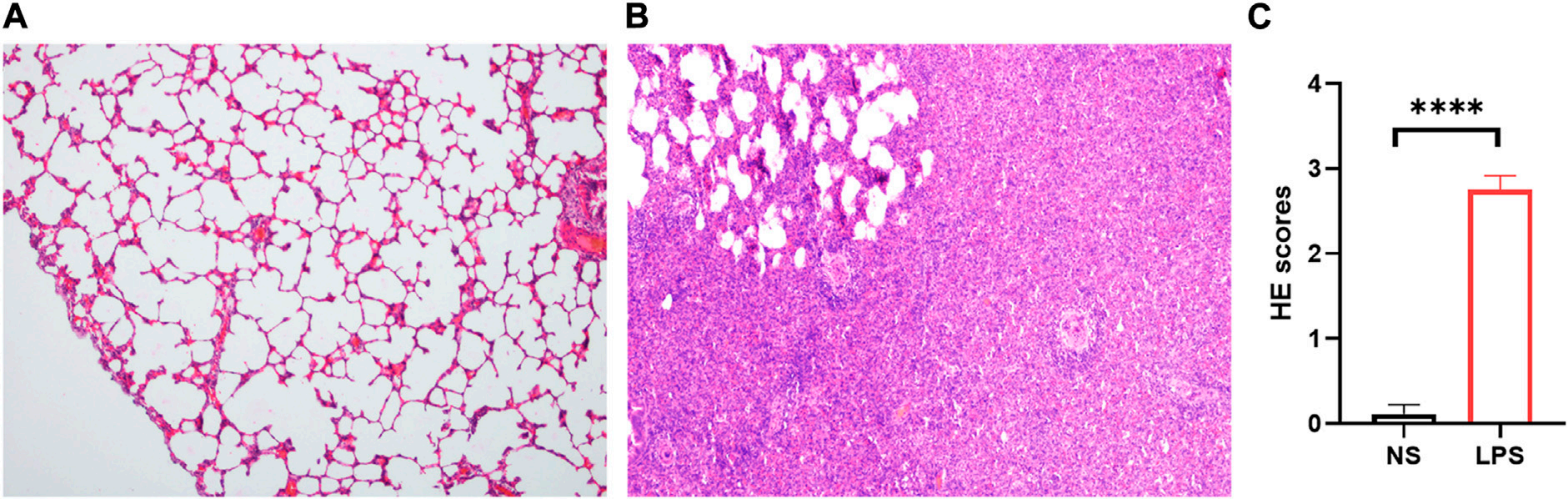
H&E staining of control and LPS-induced ARDS rats. **(A)**. Control group, magnification ×100. **(B)**. LPS group, magnification ×100. **(C)**. HE scores of the control and LPS groups.

LPS-induced ARDS rats showed marked thickening of the alveolar septa, interstitial and alveolar infiltration with inflammatory cells, hyaline membranes, alveolar haemorrhage, and focal collapse of alveolar spaces, while the control group showed normal microscopic aspects of the lung (Figures 3A, B). The LPS-induced ARDS group possessed higher ARDS scores than the control group (Figure 3C).

### The LPS-induced ARDS group had significantly higher counts of megakaryocytes (MKs) in the lung

**FIGURE 4:**
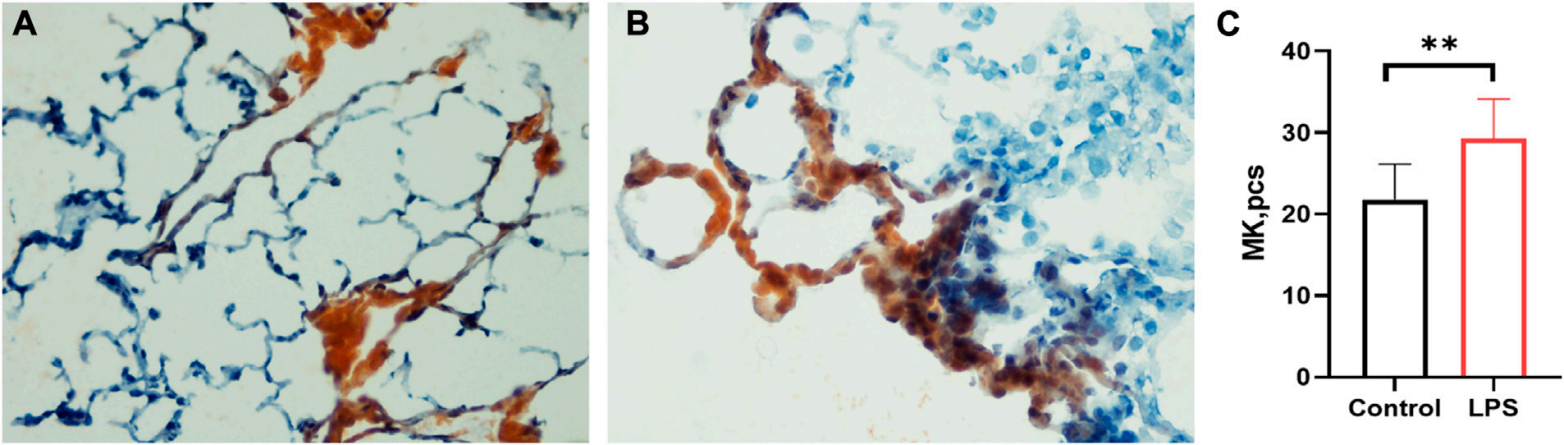
Lung megakaryocyte staining and counts. **(A)**. Lung megakaryocyte staining by AchE in the control group. **(B)**. Lung megakaryocyte staining by AChE in the LPS-induced ARDS group. **(C)**. Lung megakaryocyte differences in the control group and LPS-induced ARDS group. (magnification, ×400).

Lung megakaryocytes can produce platelets, so we detected MK counts in the lung. The LPS-induced ARDS group (Figure 4B) had significantly higher counts of MKs than the control group (Figure 4A) (LPS *versus* control group: 29.28 ± 4.862 *versus* 21.83 ± 4.319, *p* = 0.0034) (Figure 4C). The data were consistent with the high level of MK in the lungs of COVID-19 patients.

### The LPS-induced ARDS group showed inhibited production of platelets

**FIGURE 5:**
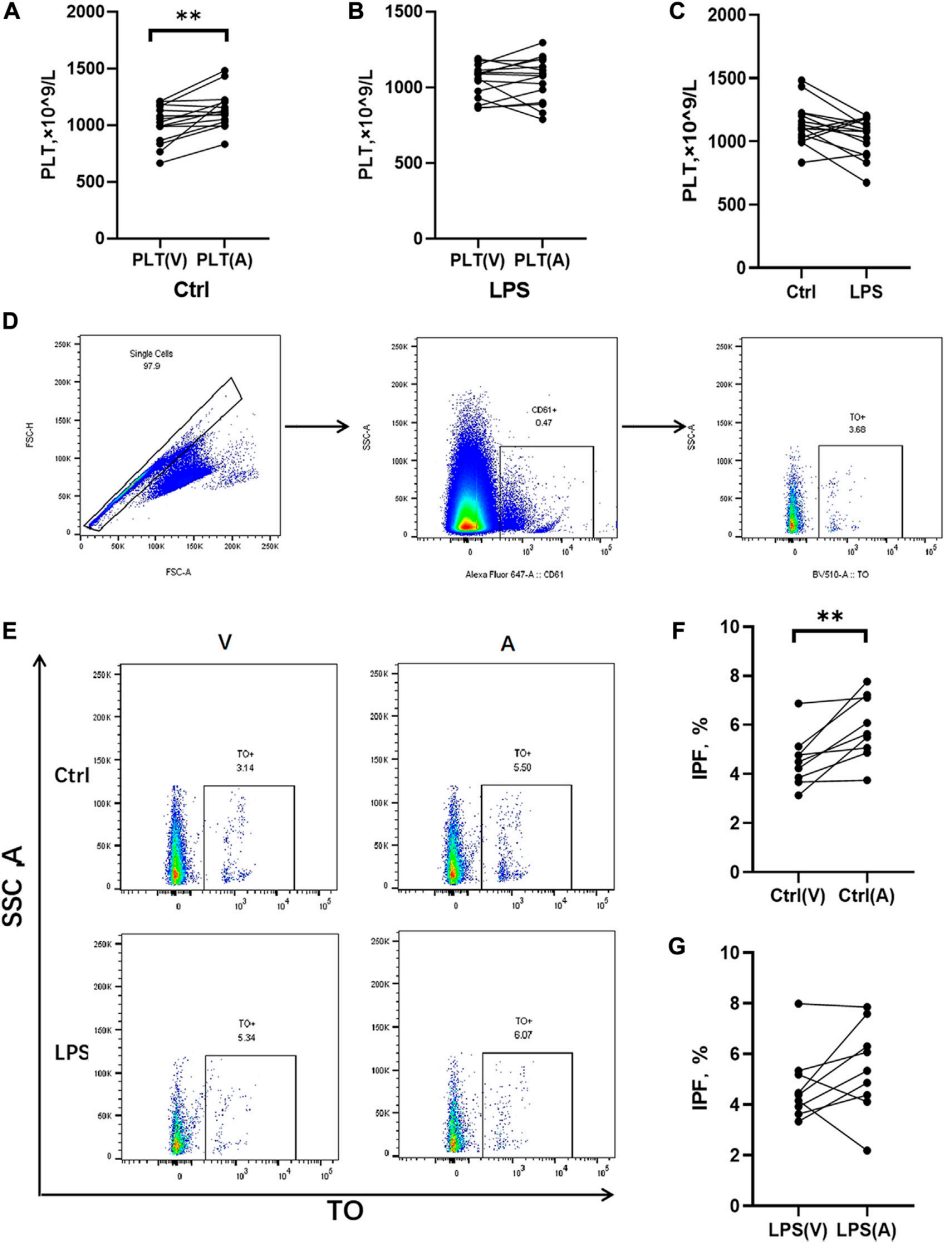
Platelet counts and IPF in prepulmonary and postpulmonary blood of the LPS-induced ARDS groups and the controls. **(A)** Prepulmonary and postpulmonary platelet counts in the control group; **(B)** Prepulmonary and postpulmonary platelet counts in the LPS-induced ARDS group, PLT(V): Prepulmonary platelet counts; PLT(A): postpulmonary platelet counts. **(C)** The peripheral blood platelet counts in the LPS-induced ARDS and control groups. **(D,E)** The strategy for gating cells in the process of analysing the immature platelet fraction (IPF). Briefly, the single cells were gated from all the cells in the prepulmonary and postpulmonary blood, and then CD61^+^ cells were gated prior to obtaining the IPF with TO+. **(F)** Prepulmonary and postpulmonary IPF in the control group; **(G)** Prepulmonary and postpulmonary IPF in the LPS-induced ARDS group. *p < 0.05, **p < 0.01.

The rats treated with LPS at 48 h, blood samples were obtained for counting platelets in the abdominal aorta (postpulmonary) and inferior vena cava (prepulmonary). In the control group, the postpulmonary platelets were significantly higher account than the prepulmonary platelets (Figure 5A), whereas the LPS-induced ARDS group had no significant difference in platelets between the prepulmonary and postpulmonary (Figure 5B). The peripheral blood platelet counts in the LPS-induced ARDS were significantly lower than control group (Figure 5C). To determine the possible reason for this result, we further detected the immature platelet fraction (IPF) in both groups, which was the precursor of platelets. CD61 was applied to mark platelets and then gated IPF with TO + conditions (Figure 5D). There was a significant difference of IPF between the prepulmonary and postpulmonary blood samples in the control group (Figures 5E, F). However, the LPS-induced ARDS group showed no significant change between prepulmonary and postpulmonary (Figures 5E, G). The above data indicated that the lung generated more IPF in the control group; in other words, the production of platelets in LPS-induced ARDS was inhibited.

## Discussion

In this study, we confirmed the existence of thrombocytopenia in COVID-19 patients, and the level of platelets was related to severity and survival. Moreover, we found that LPS-induced ARDS could inhibit platelet production in lungs of rats, which indicated that lung damage in COVID-19 may reduce platelet production in the lung. The mechanism of thrombocytopenia should be complex. The mechanisms behind thrombocytopenia in different severities of COVID-19 infection are likely due to multiple factors.

Thrombocytopenia may be caused by excessive platelet consumption, destruction by the immune system, or low production. It is well noticed that platelet-rich thrombi in pulmonary, liver, kidney, and heart microvessels have a high incidence in most autopsy cases (Wang et al., 2018) (Hirahara et al., 2019) (Kurtul and Ornek, 2019) (Rapkiewicz et al., 2020), resulting in increased platelet consumption and decreased platelet count. Borczuk et al. (2020) also found a high frequency of thrombi, especially platelet thrombi, from 68 autopsies from three institutions in heavily hit areas of Italy and New York City. The mechanism is thought to be that SARS-CoV-2 destroys the integrity of the vascular endothelium by binding with ACE2 and promotes platelet activation and thrombosis by binding with platelets, resulting in increased platelet consumption and decreased platelet count (Varga et al., 2020; Perico et al., 2022); however, one histological examination did not find this evidence (Edler et al., 2020). Excess activation of thrombocytes by virus-triggered immune complexes may be another mechanism. Cases of immune-mediated heparin-induced thrombocytopenia (HIT) have been reported (Tran et al., 2020; Pascolini et al., 2021; Mohammed et al., 2022), which means that destruction of platelets is another cause of thrombocytopenia.

Aberrant biogenesis induced by infection of bone marrow by SARS-CoV-2 may be another reason for the lower level of platelets. Possible mechanisms are likely that SARS-CoV-2 invades bone marrow and infects progenitor cells, therefore affecting thrombocyte production from megakaryocytes by inhibiting growth and inducing apoptosis (Yang et al., 2005). However, Roncati et al. (2020) reported a marked increase in naked megakaryocyte nuclei in the bone marrow and lungs from serious COVID-19 patients. It is quite interesting to find a high density of alveolar megakaryocytes in SARS-CoV-2-positive deceased patients, reported by independent research groups from Europe, the United States, and Latin America on the basis of autoptic investigations (Fox et al., 2020; Carsana et al., 2020; Duarte-Neto et al., 2020). In addition, COVID-19 patients have elevated plasma levels of thrombopoietin, a well-known megakaryocyte growth factor (Manne et al., 2020). These data indicated that COVID-19 infection induced increased megakaryocyte production in bone and high levels of megakaryocytes in the lung, blood, and heart. The possible role of the increased MK number might be compensation for the lower level of platelets; however, we wondered whether the increased MK number could produce enough platelets.

As we know, platelets are released from MK, and the lung has been found to be a very important organ for platelet production since 1937 (Howell and Donahue, 1937; Lefrancais and Looney, 2019). Direct evidence is the imaging of the lung microcirculation in mice (Pascolini et al., 2021). Lefrancais E et al. confirmed that many megakaryocytes (MKs) circulate through the lungs, where they dynamically release platelets (Borges et al., 2017). The lung contribution to platelet biogenesis is substantial, with approximately 50% of total platelet production or 10 million platelets per hour. Lungs, as a factory for platelet production, have been confirmed by human experiments, showing higher platelet counts in the pulmonary venous circulation than in the arterial side (Kallinikos-Maniatis, 1969).

Damage to lung tissue reduces the pulmonary capillary bed, and the microenvironment for platelet production is altered. It is reasonable to speculate that lung disease may affect the pulmonary megakaryocyte fragmentation process and thus reduce the level of platelets in the peripheral circulation. Platelet survival time has been found to be reduced in patients with chronic obstructive airway disease and pulmonary hypertension (Steele et al., 1977). Platelet abnormalities (Borges et al., 2017) have been reported in patients with several pulmonary diseases, such as cystic fibrosis, asthma, pulmonary tuberculosis, and pulmonary hypertension, but whether platelet biogenesis in the lungs of those patients is affected remains unknown. It was found that lung damage induced by high oxygen concentrations leads to significantly lower peripheral platelets in rats and no difference in platelet count (prepulmonary *versus* postpulmonary), while platelet level was significantly higher in postpulmonary of healthy rats (Yang et al., 2003). Xiao da et al. (2006) also found that postpulmonary blood had a lower MK count and higher platelet count in healthy rats, but high oxygen concentration damaged animals showed no such differences in either MK or platelet count, and peripheral platelets were significantly lower than controls. It is the only report referring to impaired platelet production by lung injury, as we know.

The main pathology of COVID-19 in the lung is DAD, and it is reasonable to consider that thrombocytopenia is partly due to changes in the pulmonary microenvironment, which is important for platelet biogenesis. SARS-CoV-2 infection is always accompanied by thrombocytopenia in severe patients (Hon et al., 2003; Yang et al., 2004). Valdivia-Mazeyra found that pulmonary megakaryocytes were increased in patients with DAD regardless of the cause (Valdivia-Mazeyra et al., 2021). Lung biopsy specimens from 21 patients with diffuse alveolar damage demonstrated increased microvascular CD61þ megakaryocytes, reflecting injury to the pulmonary microvasculature and causing impaired megakaryocyte fragmentation (Mandal et al., 2007). Damage to the lung microvasculature may also be a reason for thrombocytopenia commonly noted in ARDS. In COVID-19, thrombocytopenia caused by excessive consumption and destruction leads to a compensatory increase in thrombopoietin, and bone marrow megakaryocytes increase with focal clustering for active platelet production. Pulmonary and heart megakaryocytes were subsequently increased (Rapkiewicz et al., 2020). However, DAD might inhibit platelet production in the lung. The compensation of platelet biogenesis could not be realized. In this study, we demonstrated that LPS-induced ARDS rats exhibited no increase in platelet count in postpulmonary blood compared with prepulmonary blood, which was significantly higher in postpulmonary blood in controls. This indicates that there was no significant net platelet production in the pulmonary capillary bed. To confirm our supposition, we further detected IPF in prepulmonary and postpulmonary blood. There was a significant increase of IPF in postpulmonary blood than prepulmonary in the control group, but the LPS-induced ARDS group had no changes, indicating that more IPF was produced in the control group. In addition, MK staining showed that LPS-induced ARDS rats had higher MK counts, which means that MK was trapped in the lung and released fewer platelets, providing evidence of lung platelet biogenesis obstruction. As LPS-induced ARDS is imitated with COVID-19-induced ARDS pathology, this result may supply new evidence that illustrated abnormal production of platelets also participates in the pathogenesis of thrombocytopenia in COVID-19. The findings supported our hypothesis that the process of MK fragmentation to release platelets was impaired in the injured lung. To the best of our knowledge, this is the first report that ARDS induce obstruction of platelet production in lung.

Besides of the failure to increase the IPF, more possible mechanisms maybe contribute to the inhibition of platelet. 1) In LPS-induced ARDS, lung capillary bed was damaged, leading to megakaryocyte retention (Figure 4) which may be an important reason for the lack of increase in IPF. As a result, platelets release decreased. 2) As we know, bone marrow and the lung are two important organs for producing platelets. The multifunctional hematopoietic stem cells in the bone marrow differentiate into primitive megakaryocytes, which mature to produce platelets. Study have found that circulating platelets in patients with severe COVID-19 contain SARS-CoV-2. ACE2 is a receptor of SARS-CoV-2, and ACE2 is expressed in bone marrow (Li et al., 2020), indicating SARS-CoV-2 may directly inhibit bone marrow and further affect megakaryocytes cell production. Several studies have shown that SARS-CoV-2 may directly infect megakaryocytes (Shen et al., 2021; Zhu et al., 2022). 3) Interestingly, SARS-CoV-2 spike protein is responsible for entering host cells, and it was found that SARS-CoV-2 spike protein antibody cross-react with human thrombopoietin (TPO) through molecular simulation. TPO is an important factor in megakaryocytes maturation and differentiation. When human bodies produce SARS-CoV-2 antibodies, the antibodies also inhibit TPO, which in turn affects platelet production (Nunez-Castilla et al., 2022). The mechanism of thrombocytopenia in COVID-19 patients should be complex and much works are needed to elucidate.

PLR is commonly used as an inflammatory index. PLR represents the ratio of platelets to lymphocytes, which can reflect the characteristics of both platelets and lymphocytes. The PLR values detected in patients with different bacterial infections are different, and the PLR in patients with Gram-positive bacteria is significantly lower than that in patients with Gram-negative bacteria (Djordjevic et al., 2018). PLR could predict the mortality rate within 90 days in patients with acute exacerbation of COPD (Kumar et al., 2017). The PLR was significantly higher in neonatal early-onset septicaemia patients than in healthy controls (Arcagok and Karabulut, 2019). In addition, PLR has significance in a variety of tumour diseases, respiratory diseases, cardiovascular diseases, rheumatic diseases, and so on (Gasparyan et al., 2019; Wang et al., 2018; Hirahara et al., 2019; Kurtul and Ornek, 2019). Platelets can promote lymphocyte adhesion and recruitment, regulate T lymphocyte activation, and stimulate B lymphocyte proliferation and antibody production; conversely, lymphocytes can regulate platelet aggregation and secretion (Li, 2008). This study also compared the levels of PLR among different severity groups and calculated the optimal threshold of PLR. Our study found that the PLR of patients with severe COVID-19 was significantly increased, which was consistent with the findings of Yang A. P. et al. (2020) and Qu et al. (2020) (Wang X. et al., 2020), although the PLR threshold was relatively higher than that of Yang and Qu. The area under the ROC curve of PLR is greater than 0.7, which indicates that PLR has good value for the prognosis of patients with COVID-19. When PLR is greater than 248.5, attention should be given to the risk of aggravation and even death.

In conclusion, the platelet count and PLR could represent disease severity and predict prognosis in COVID-19. Production obstruction in the lung might be one mechanism of the lower level of platelets.

Limit: We recognize the limitations of our clinical observations and animal models. The records may be incomplete with possible variations in the data, especially during the special period of the first outbreak of COVID-19. Because of biosafety limitations, the SARS-COV-2-induced DAD model was substituted by the LPS-induced ARDS rat model.

## Materials and methods

### Clinical studies

This study only retrospectively reviewed a cohort of patients who were admitted to Wuhan Third Hospital in Guanggu District, which was a referral centre for local COVID-19 patients diagnosed in the public health centre from January 28 to March 20 in 2020 in Wuhan, China. All patients were confirmed by SAS-CoV-2 testing before admission. The test was performed using nasopharyngeal swabs and real-time reverse transcriptase PCR as described previously. The severity of pneumonia in these patients was assessed according to the Guidelines for Diagnosis and Management of COVID-19 (sixth edition, in Chinese) issued by the National Health Commission of China (The National Health Commission of the People’s Republic of China main website). https://www.nhc.gov.cn. Accessed 18 February 2020. Briefly, four groups were defined according to disease severity: 1) The mild type findings include mild clinical symptoms with no imaging performance; 2) The common type includes fever, respiratory symtoms and imaging findings of pneumonia;3) The severe type meets any of the followings: 1) respiratory distress, RR ≥ 30 times/min; 2) Oxygen saturation (SpO_2_) < 93% at rest; 3) arterial partial pressure of oxygen (PaO_2_)/fraction of inspiration oxygen (FiO_2_) ≤ 300 mmHg; 4) The critical type meets any of the followings: 1) respiratory failure needs mechanical ventilation; 2) shock; 3) combined with other organ failure, patients need ICU monitoring and treatment. Exclusion criteria: Patients with haematological disorders such as leukemia, aplastic anaemia, thrombocytopenia due to genetic or rheumatic immune factors, drug-associated thrombocytopenia, and patients undergoing chemoradiotherapy. All 157 cases in our ward were retrospective analysed. Due to the serious epidemic situation in China at that time, the data of some patients were incomplete, so, these cases (61 cases) were excluded. One case was excluded because of having a hepatoma undergoing chemotherapy. Finally, total 95 patients were selected from hospitalized patients. The study was approved by the Ethics Committee of Wuhan Third Hospital, and the requirement for written informed consent was waived because of the retrospective nature of the study.

### Experimental animals and ARDS modelling

In total, 20 clean-grade 6-week-old male Sprague Dawley rats with an average weight of 200 ± 10 g were purchased from Shanghai SLAC Laboratory Animal Co., Ltd. The rats were maintained (four rats per cage) at the Rodent Testing Center of East China Normal University. Rats were raised under specific pathogen-free laboratory conditions at a constant ambient temperature (23°C–25°C) and humidity (40%–50%) with free access to food and water under a normal 12-hour light/dark cycle. The experiments were carried out after 1 week of adaptive feeding. The animal experiments were approved by the Experimental Animal Welfare and Ethics Committee of Putuo Hospital of Shanghai University of Chinese traditional Medicine (number: 17-08). The ARDS model was established as described in previous reports (Cleary et al., 2019; Huang et al., 2019) (Hou et al., 2014; Wei and Wang, 2017). Briefly, rats were randomized to two experimental groups (10 animals/group). The LPS group was induced by intratracheal instillation of LPS (*Escherichia coli* 0111: B4, 20 μg/g body weight) (Sigma Chemical, St. Louis, MO). Rats in the control group received only saline solution. The animals were confirmed by computed tomography (CT) and Oxygenation index (PaO_2_/FiO_2_) analysis. The animals were exsanguinated after 48 h, and lung tissue and blood were collected for further analysis.

### Lung computed tomography

Animals were anaesthetized (45 mg/kg, 3% sodium pentobarbital solution) and checked by computed tomography (GE optima 540) to confirm the establishment of ARDS after LPS instillation at 16 h. The images were viewed by an Onis 2.5 Free Edition, with window settings optimized for the assessment of the lung parenchyma (width 2000 HU; level − 500 HU).

### Oxygenation index (PaO2/FiO2) analysis

After LPS treated for 24 h, the arterial blood was obtained from carotid artery without oxygen therapy. The arterial blood was analysed by an automatic blood cell analyser (Sysmex XE) (Fu et al., 2017).

### Platelet counts

Peripheral blood was collected in an anticoagulant tube by snipping the tail of the rats. An automatic blood cell analyser (Sysmex XN) was used to detect rat peripheral platelets (Lee and Kang, 2016).

### Flow cytometry analysis

Blood from the abdominal aorta (postpulmonary) and inferior vena cava (prepulmonary) was drawn with a venous blood collection needle and stored in an anticoagulant tube for the timely detection of immature platelet fraction. Cells from rat blood were subjected to flow cytometry analysis on a multicolour flow cytometer (BD, Celesta). CD61 antibody was purchased from Biolegend, and thiazole orange (TO) was obtained from MCE. All data were analysed with FlowJo software (Treestar, Ashland, OR, United States).

### Lung histology

The left base lobe of each rat lung was obtained and fixed with 10% formalin buffer overnight. Lung tissues were embedded in paraffin and sectioned followed by HE staining, including conventional dewaxing, haematoxylin staining, ethanol differentiation, eosin staining, dehydration, and neutral balsam mounting. Histologic examination for evidence of lung damage was carried out under light microscopy.

### Lung megakaryocyte identification

The right upper lobe of the lung was removed by surgery, fixed with tissue freezing medium (Tissue-Tek^®^ O.C.T. Compound, Number 4583) and stored at −80°C. Frozen sections (6 μm) were labelled with AchE (Sigma Chemical Co., Saint Louis, United States) and counterstained with haematoxylin to identify megakaryocytes as previously described (Yang et al., 2003). In brief, after washing the slides with distilled water, they were stained with AChE at room temperature for 3 hours. Then, the slides were counterstained with haematoxylin for 3–5 min, rinsed with distilled water, and routinely dehydrated and fixed. Lung MKs were counted in 20 random fields from three lung sections per animal at ×40 magnification. Fields with large bronchi or vessels were excluded.

### Statistics

SPSS 21.0 was used to analyse the data. Continuous variables and categorical variables are expressed as the mean ± standard deviation and n (%). The rank sum test of measurement data (Kruskal‒Wallis test), one-way analysis of variance (ANOVA) statistical method, and chi-square test of enumeration data were applied to analyse patient characteristics. The Bonferroni method was used for groups’ (mild, common, severe, critical) multiple independent sample comparisons. The Pearson chi-square test was used to analyse the correlation between PLTlow and death, and the OR value was calculated. By analysing the ROC curve of PLR and calculating the area under the curve (AUC), the optimal threshold, specificity, and sensitivity of PLR were determined. Binary logistic regression analysis was conducted to determine the influence of age, gender, and other significant factors. Two-sample *t*-test was used to analyse PaO2/FiO2. Paired *t*-test was used to analyse platelet counts and IPF in prepulmonary and postpulmonary blood of animals. Normal distribution data were expressed as mean ± standard deviation, and non-normal distribution data were represented by the median (interquartile range). *p* < 0.05 was considered statistically significant. GraphPad 8.4 was used for drawing.

## Data Availability

The original contributions presented in the study are included in the article/Supplementary Material, further inquiries can be directed to the corresponding authors.
