# Supplementary material for: Biogenesis aberration: One of the mechanisms of thrombocytopenia in COVID-19
Source: Front Physiol. 2023 Mar 20;14:1100997. doi: 10.3389/fphys.2023.1100997 (PMC10067878; doi:10.3389/fphys.2023.1100997)
Supplement: Supplementary file 1 [file Table1.DOCX]

**Supplementary Materials**

**Table S1. The adjusted odds ratio of PLTlow, PLRlow**

|  | *P* | Adjusted odds ratio (ORa) | 95%CI |
| --- | --- | --- | --- |
| PLTlow | 0.038 | 1.013 | 1.001~1.026 |
| PLRLow | 0.024 | 0.994 | 0.989~0.999 |

Abbreviations: PLTlow is the lowest platelet value during hospitalization. PLRlow is the PLR corresponding to the lowest platelet value during hospitalization.


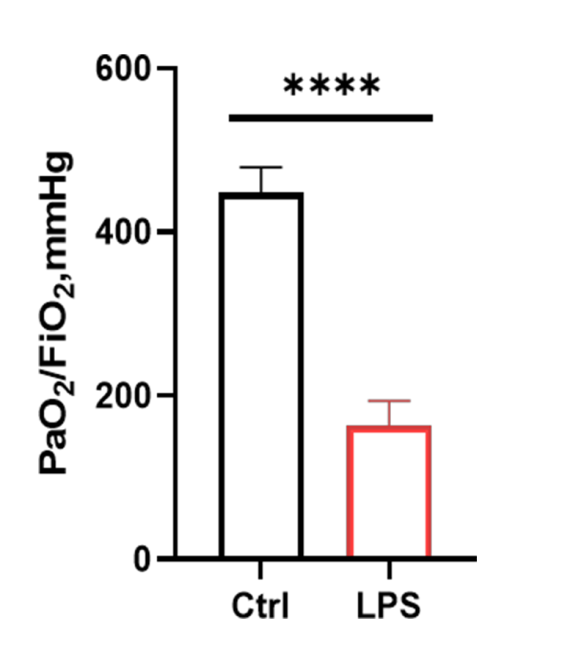


**Fig.S1. The PaO_2_/FIO_2_ of control and LPS-induced ARDS rats.** The lung dysfunction was determined by the oxygenation index (PaO2/FIO2). *****P* < 0.0001.
