# Supplementary figures and images for: Biogenesis aberration: One of the mechanisms of thrombocytopenia in COVID-19
Source: Front Physiol. 2023 Mar 20;14:1100997. doi: 10.3389/fphys.2023.1100997 (PMC10067878; doi:10.3389/fphys.2023.1100997)

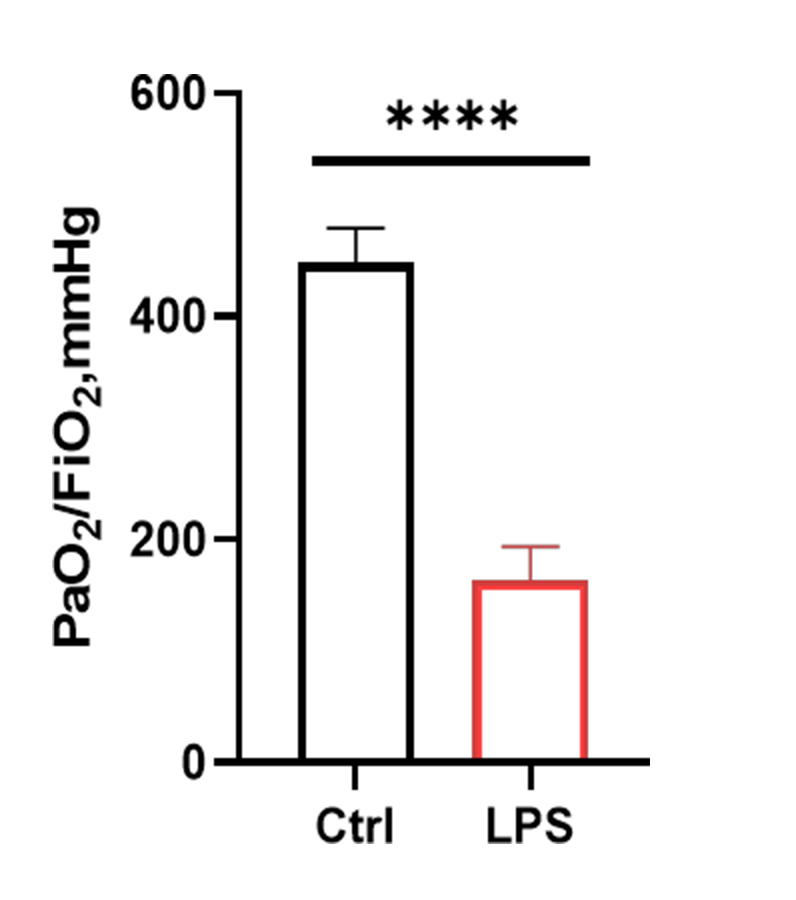

Supplement: Supplementary file 2 [file Image1.TIF]
